# Role of Main RNA Methylation in Hepatocellular Carcinoma: N6-Methyladenosine, 5-Methylcytosine, and N1-Methyladenosine

**DOI:** 10.3389/fcell.2021.767668

**Published:** 2021-11-30

**Authors:** Yating Xu, Menggang Zhang, Qiyao Zhang, Xiao Yu, Zongzong Sun, Yuting He, Wenzhi Guo

**Affiliations:** ^1^ Department of Hepatobiliary and Pancreatic Surgery, The First Affiliated Hospital of Zhengzhou University, Zhengzhou, China; ^2^ Key Laboratory of Hepatobiliary and Pancreatic Surgery and Digestive Organ Transplantation of Henan Province, The First Affiliated Hospital of Zhengzhou University, Zhengzhou, China; ^3^ Open and Key Laboratory of Hepatobiliary and Pancreatic Surgery and Digestive Organ Transplantation at Henan Universities, Zhengzhou, China; ^4^ Henan Key Laboratory of Digestive Organ Transplantation, Zhengzhou, China; ^5^ Department of Obstetrics and Gynaecology, The Third Affiliated Hospital of Zhengzhou University, Zhengzhou, China

**Keywords:** hepatocellular carcinoma, RNA methylation, M6A, m5C, m1A

## Abstract

RNA methylation is considered a significant epigenetic modification, a process that does not alter gene sequence but may play a necessary role in multiple biological processes, such as gene expression, genome editing, and cellular differentiation. With advances in RNA detection, various forms of RNA methylation can be found, including N6-methyladenosine (m6A), N1-methyladenosine (m1A), and 5-methylcytosine (m5C). Emerging reports confirm that dysregulation of RNA methylation gives rise to a variety of human diseases, particularly hepatocellular carcinoma. We will summarize essential regulators of RNA methylation and biological functions of these modifications in coding and noncoding RNAs. In conclusion, we highlight complex molecular mechanisms of m6A, m5C, and m1A associated with hepatocellular carcinoma and hope this review might provide therapeutic potent of RNA methylation to clinical research.

## Introduction

Hepatocellular carcinoma (HCC) is a common global disease. It has a poor prognosis and has become the third cause of cancer death (Couri and Pillai, 2019; Hua et al., 2019; Huang et al., 2021). Although medical technology has significantly improved in recent years, 5-years survival rates of patients remain low (8.5%) (Cai et al., 2019; Zhou et al., 2020). Mortality due to HCC remains high for several reasons. On one hand, clinical symptoms in the early stage are generally displayed uncharacteristic and the lack of effective diagnostic biomarkers, so many patients are easily misdiagnosed. On the other hand, as a result of affluent blood supply in the liver, tumor cells frequently proliferate at a growing rate and distant metastasis tends to appear in the early stage of cancer. Therefore, HCC seriously threatens human health and well-being and is seen as a tough challenge in clinical study. Emerging research is exploring the understanding of pathogenesis in HCC to prevent the dilemma of poor prognosis. Previous reports have showed that the leading pathogenic factor is chronic infection with virus, such as hepatitis B virus and hepatitis C virus (Koshiol et al., 2021; Teng et al., 2021). Nevertheless, other molecular mechanisms involved in proliferation, invasion, metastasis, and chemoresistance in HCC remain unknown. Consequently, it is crucial to further investigate the complex mechanisms of tumorigenesis and tumor progression to discover novel makers and identify therapeutic targets.

RNA methylation is commonly regarded as posttranscriptional modification with multiple forms (Chen et al., 2019; Kagra et al., 2021). Although epigenetic modification of RNA has been documented over several decades (Cohn, 1960; Dubin and Taylor, 1975; Perry et al., 1975), our understanding of its biological functions is still limited. Recent research demonstrates that RNA modification may impact RNA metabolism, splicing, stability, and translation (Xue et al., 2020; Nombela et al., 2021), which distinctly influence gene expression. Thus, the effect of RNA methylation is gradually attracting broad attention in a broad array of specialties. For example, numerous investigations verified that m5C methylation in the 3′-UTR of mRNA increases translation efficiency (Schumann et al., 2020). Occurrence of RNA methylation ordinarily requires the participation of a large number of specific proteins called RNA-modifying proteins (RMPs), containing “writers,” “erasers,” and “readers” (Frye et al., 2018; Patil et al., 2018). “Writers” are a group of enzymes that catalyze methylation. In contrast, “erasers” are able to remove the decorate of methylation in RNA (Torres and Fujimori, 2015; Shi et al., 2019). “Readers” are a variety of proteins that recognize methylation sites catalyzed by “writers” and bind these sites to form complexes to affect the functions of RNA (Pozner et al., 2018; Grimanelli and Ingouff, 2020; Guo et al., 2020).

A prior survey revealed that abnormal regulation of these RMPs would give rise to incidence of various malignant tumors (Pan et al., 2018; Lan Q. et al., 2019; Zhuang et al., 2020; Li Y. et al., 2021). For instance, catalysis of m6A modification is mediated by methyltransferase-like 3 (METTL3), which is expressed at a high level in colon cancer. Previous evidence showed that METTL3 promoted miRNA-1246 upregulation and induced metastasis in colon cancer (Peng et al., 2019). In bladder cancer, YBX1 obviously emerged the appearance of overexpression than normal tissue. YBX1 is an RMP “reader” in m5A modification, and might advance the expression of the multidrug resistance-1 (MDR-1) gene to decrease sensitivity to chemotherapy drugs (Yamashita et al., 2017). Additionally, downregulation of DKC1 was found in breast cancer and gave rise to the impairment result of hTR stabilization (Montanaro et al., 2006). Similarly, overexpression of ALKBH3, methylating affluent m1A modification, ordinarily predicts a dismal prognosis in Hodgkin lymphoma (Yin et al., 2020). Other reports showed that PTR can lengthen survival time during stage M1a of non-small cell lung carcinoma (Li et al., 2019; Li H. et al., 2020), and was expected to be incorporated into promising therapeutic strategies for diagnosing patients with evolving ipsilateral pleural dissemination. Various experiments were testified that regulators of RNA methylation, like m6A, m5C, and m1A, participated in essential biological process for diverse cancers (Li C.-L. et al., 2020). However, discussions that effect of these regulatory factors in RNA methylation related to pathogenesis of HCC, are constricted in clinical study. Accordingly, in this review we illustrate functional consequences of m6A, m5C, and m1A in diverse RNAs. Cooperatively, we focus on targeting RMPs for clinical treatment in HCC in anticipation of providing patients with more promising overall survival and brighter futures.

## RNA Methylation

Methylation refers to epigenetic transformations to influence gene expression but does not alter gene sequence, which can be mainly found in DNA, RNA, and protein (Wang X. et al., 2020; Hop et al., 2020; Anton and Roberts, 2021). As approaches to detect RNA modifications improve, RNA methylation began to broadly draw public notice. Multiple functional effects of modification of RNAs were further discussed, and the role of RNA methylation correlative with a variety of cancers also gradually become clearer. We briefly generalize these forms of methylating modification as follows.

### N6-Methyladenosine

m6A is methylated adenosine at the nitrogen-6 position and was identified as a posttranscriptional modification in 1974 (Desrosiers et al., 1974). Previous survey has considered that m6A modifications are one of the most extensive methods of RNA methylation in mammals. It was estimated that m6A methylation was approximately present on a quarter of mRNAs (Meyer and Jaffrey, 2017; Chen et al., 2018). As approaches of detecting m6A is distinctly preferred, the utilization of ultraviolet crosslinking step realized the new outcome, identifying m6A positions at single-nucleotide resolution. Specific locations of m6A modification are detected, such as 3′ untranslated regions (3′-UTRs) (Dominguez et al., 2018), long internal exons, intergenic regions, introns, and 5’ UTRs. METTL3 was first identified in the occurrence of m6A modification. METTL3 functions as the regulator mediating the export of mRNA by interacting with Per2 and Arntl. In addition, METTL3 can recruit eukaryotic translation initiation factor eIF3 to directly regulate translation flexibly (Lin et al., 2016; Choe et al., 2018). The other “writers” of m6A include METTL14, Wilms tumor 1-associated protein (WTAP), and RNA-binding motif protein 15 (RBM15) (Liu et al., 2014; Wen et al., 2018). Accordingly, “readers” of m6A are primarily proteins in the YT521-B homology (YTH) domain family and include YTHDF1, YTHDF2, YTHDF3, YTHDC1, and YTHDC2. Accumulating evidence demonstrates that promotion of translation can positively modulate the effect of YTHDF1. YTHDF3 accelerates protein synthesis by binding YTHDF1 to mediate ribosomal proteins (Shi H. et al., 2017; Li et al., 2017). In m6A methylation, fat mass and obesity-associated protein (FTO) and alkB homologue 5 (ALKBH5) are considered “erasers”. FTO binds to introns of nascent mRNA molecules to modulate the biological process of splicing in mRNA (Bartosovic et al., 2017). Similarly, multiple reports also confirmed that ALKBH5 is a pivotal factor to participate in mRNA splicing (Zheng et al., 2013).

### 5-Methylcytosine

m5C is defined as the accession of methyl group on the fifth carbon atom of cytosine (Motorin et al., 2010; Huang et al., 2019). Abundant m5C occurs in a variety of RNAs, including mRNA, tRNA, rRNA, viral RNA, vault RNA, and lncRNA. In humans, m5C is introduced by NSUN family members and DNA methyltransferase 2 (DNMT2). NSUN2 methylates primarily tRNA and mRNA. The defined regions of tRNA are the variable loop and leucine at the wobble position (Hussain et al., 2013; Khoddami and Cairns, 2013). In mRNA, the specific sites of catalysis by NSUN2 are the region near the start codon and the noncoding 3 UTR. Distribution of NSUN2 is unique, because of converting altogether with different alteration of cell division cycle. NSUN2 can be found at the nucleolus in G1 phase, whereas it is located in the region between the nucleolus and nucleoplasm in S phrase. NSUN2 starts to gradually appear in the cytoplasm in G2 and M phase (Motorin et al., 2010). It was reported that centrioles could be detected abundant depositions of NSUN2 during M phrase. Previous study declared that NSUN2 played an indispensable role in phosphorylation, protein synthesis, cell cycle progression, and epidermal differentiation and tumorigenesis. NSUN4 and NSUN5 primarily catalyze methylation modification in 25s rRNA. NSUN4 protein is frequently found in mitochondria, but NSUN5 is distributed in the nucleolus. Overexpression of NSUN5 promotes synthesis of survival protein to enhance the response to oxidative stress (Schosserer et al., 2015). Most NSUN1 factors are detected in the nucleolus, although a few are detected in the cytoplasm. NSUN1 was found to participate in malignant invasion, cell cycle progression, and formation of chromatin (Sharma et al., 2013). NSUN3 and DNMT2 methylate tRNA and are distributed in mitochondria and cytoplasm, respectively. Numerous experiments indicated that DNMT2 has a critical influence in tumorigenesis, protein synthesis, cell differentiation, and HIV-1 RNA replication (Dev et al., 2017). The “erasers” of m5C methylation are primarily TET family members. TET1 catalyzes the removal of methylation in coding and non-coding RNAs. In addition, several reports suggested ALYREF recognizes and binds the methyl group catalyzed by NSUN2 in mRNA. ALYREF and NSUN2 together promote the transport of mRNA and increase the efficiency of nuclear-cytoplasmic shuttling (Shi M. et al., 2017). Upregulated YBX1 was observed in the cytoplasm and exerted a positive effect on mRNA stabilization, embryogenesis, and tumorigenesis (Yang et al., 2019).

### N1-Methyladenosine

m1A is methylation at the N1 position of adenosine and is capable of altering RNA secondary structure. A previous study identified m1A in tRNA, rRNA, mRNA, and mitochondrial RNA. Affluent m1A modification is observed in tRNA and rRNA, while the level of m1A remains low in mRNA. The occurrence of m1A methylation in mRNA is represents a six-fold reduction compared to that of m6A methylation (Dominissini et al., 2016); however, m1A can be found in the coding sequence (CDS), 5′-UTR (Li et al., 2016), and 3′-UTRof mRNA. Emerging survey suggested method of m1A modification involving in protein synthesis, which improved the efficiency of translation by inhibiting binding of the releasing factor. In contrast, when m1A methylation occurs in the region of mRNA CDS, translation is suppressed to some degree. TRMT10C and TRMT61B serve as “writers” to participating in catalyzing m1A at position 9 and 58 of tRNA (Chujo and Suzuki, 2012). ALKBH3 and ALKBH1 not only demethylate the reversible modification of m6A, but are found to remove m1A (Liu F. et al., 2016). ALKBH3 can function as a repair enzyme to restore N-methylated bases. Recent investigations clarified that demethylation by ALKBH3 might improve the efficiency of translation. Therefore, silencing of ALKBH3 may have the effect of impeding protein synthesis by enhancing the level of m1A in tRNA. Moreover, ALKBH3 was regarded as prostate cancer antigen-1 (PCA-1) (Shimada et al., 2009; Yamato et al., 2012). Upregulation of ALKBH3 was observed in a variety of cancers, which stimulated angiogenesis and inhibited apoptosis in prostate cancer and pancreatic cancer patients. The present study suggested the function of m1A58 may result in decreased translation initiation. When ALKBH1 demethylates m1A, the elongation phase of translation might be impacted through reduced tRNA usage in protein synthesis (Haag et al., 2016; Kawarada et al., 2017).

## Functional Consequences of RNA Methylation

RNA methylation takes place in various RNAs, which give rise to different outcomes to influence RNA function (Gilbert et al., 2016). The detailed functional consequences associated with modifications m6A, m5C, and m1A in RNAs are presented in Table 1.

**TABLE 1:** The modification results of m6A, m5C, and m1A methylation in various RNA.

RNA type	Regulators	Modification type	Functional consequences	PMID
mRNA	YTHDF2	m6A	Enhance stability	29,476,152
mRNA	YTHDC1	m6A	Promote export	30,218,090
mRNA	ALKBH5	m6A	Promote export	23,177,736
mRNA	METTL3	m6A	Elevate translational efficiency	27,117,702
mRNA	METTL14	m6A	Elevate translational efficiency	24,284,625
mRNA	ALYREF	m5C	Promote export	28,418,038
mRNA	NSUN2	m5C	Promote transport and affect protein synthesis (promote, and inhibit)	25,063,673
tRNA	NSUN2	m5C	Enhance stability and promote survival proteins synthesis to repose stress	28,062,751
rRNA	NSUN5	m5C	Enhance stability	27,167,997
mRNA	—	m1A	Enhance stability and affect translation efficiency (promote, and inhibit)	28,230,814
tRNA	—	m1A	Promote HIV replication	29,908,293
tRNA	ALKBH1	m1A	Enhance stability	27,984,735

### Role of m6A in RNA

The stability of mRNA is mainly regulated by modification m6A. YTHDF2, an m6A “reader”, might recruit mRNA into processing bodies and participate in the process of degradation to stabilize mRNA (Wang et al., 2014; Huang et al., 2018). Numerous studies reported that YTHDC1 is involved in triggering the SRSF3 pathway to mediate dynamic splicing of precursor mRNA (Molinie et al., 2016). Furthermore, YTHDC1 promotes the export of mRNA (Roundtree et al., 2017; Lesbirel et al., 2018), recruits nuclear transport receptors, and interacts with TREX mRNA adducts. In contrast, depletion of ALKBH5 accelerates export of mRNA (Zheng et al., 2013). Nuclear export is indispensable for translation of mRNA to protein. Several reports corroborated that METTL3 and METTL14 catalyze the modification m6A in the region of the 3′-UTR in p21 mRNA and positively increase the efficiency of translation. The recruitment of DCGR8 is mediated by METTL14 in pri-miRNA to encode and regulate the level of miR-126a (Wang et al., 2014). METTL14 is also important for transcriptional elongation of chromatin, which brings about the outcome of recruiting the microprocessor complex (Nombela et al., 2021).

### Role of m5C in RNA

In mRNA, modification m5C can have a significant impact on metabolism. ALYREF was considered to enhance the efficiency of nuclear-cytoplasmic export by forming a complex with mRNA. Previous experiments confirmed that NSUN2 meditates mRNA transport, which facilitates ALYREF binding to mRNA (Yang et al., 2017). Therefore, m5C affects protein synthesis to a degree. For instance, modification m5C appears in CDS in mRNA, which impairs translation and reduces its efficiency. On the contrary, when m5C is located at the 3′-UTR, the productivity of protein synthesis is distinctly improved. Diverse locations of m5C might lead to different functional results. Moreover, the modification m5C might maintain the stability of mRNA and facilitate plant development. NSUN2 might mediate root-development–related transcripts to suppress root decay. The present survey found that NSUN2 and DNMT2 mediate m5C methylation and commonly play an essential role in stabilizing tRNA. When cells were exposed to hydrogen peroxide, NSUN2 generated survival proteins to respond to the stress (Blanco et al., 2014; David et al., 2017). DNMT maintains the stability of tRNA Asp-GTC and tRNA Gly-GCC and increases the efficiency of polypeptide synthesis (Tuorto et al., 2015). Several evidences suggest NSUN5 modulates rRNA stability under conditions of oxidative stress (Schosserer et al., 2016). NSUN4 impacts regulation of the last step of ribosomal biogenesis (Metodiev et al., 2014).

### Role of m1A in RNA

Modification m1A is found predominantly in structured regions of the 5′-UTR and near alternative start codons, indicating that m1A is significantly involved in stabilizing mRNA structure. The accomplishment of m1A methylation also exerts an indispensable effect on translation efficiency. For instance, m1A in the CDS region of mRNA has been considered to block the productivity of protein synthesis because it disrupts Watson-Crick base pairing. The presence of m1A might be vital to regulate the structural thermostability of tRNAs. It was reported that m1A together with other post-transcriptional modifications is capable of enhancing the melting temperature of tRNAs. ALKBH1 deficiency improves the cellular level of tRNA-Met to maintain the functional effect of m1A, stabilizing tRNA-Met (Liu F. et al., 2016). On the contrary, deficiency of enzymes catalyzing the achievement of m1A, have the possibility of induce thermosensitivity (Oerum et al., 2017). Moreover, m1A in tRNA-Lys was found to play an important role in reverse transcription fidelity and participate in the process of HIV replication.

## Mechanism of RNA Methylation IN HCC

Recently emerging evidence has demonstrated that RNA methylation plays a dramatic role in tumorigenesis, invasion, and migration of HCC and elucidated complex mechanisms. We present the evidence for regulators and the effect of m6A, m5C, and m1A related to initiation and progression of HCC in Table 2.

**TABLE 2:** The association of m6A, m5C, and m1A methylation in HCC.

Modification type	Regulators	Expression	Clinical characters	Function in HCC	Target	PMID
m6A	ALKBH5	Down	Favorable prognosis	Inhibit proliferation and invasion	LYPD1	32,772,918
m6A	METTL3	Up	Poor prognosis	Promote vascular invasion, and metastasis	HBXIP	33,305,825
m6A	YTHDF3	Up	Poor prognosis	promote invasion, migration, and EMT	Zeb1	32,653,519
m6A	METTL14	Down	Favorable prognosis	Inhibit invasion, migration, and EMT	EGFR/PI3K/Akt	33,380,825
m6A	YTHDF1	Up	Poor prognosis	Promote proliferation, migration, and invasion	PI3K/Akt/mTOR	34,088,349
m6A	FTO	Up	Poor prognosis	Promote initiation, metastasis, and chemoresistance	AMD1	33,783,988
m5C	NSUN2	Up	Poor prognosis and advanced TNM stage	Promote metastasis	H19	32,978,516
m5C	NSUN4	Up	Poor prognosis	—	—	32,269,723
m5C	ALYREF	Up	Poor prognosis	—	—	32,944,246
m1A	TRMT6	Up	Poor prognosis	—	PI3K/Akt	32,934,298

### m6A Links to HCC

Numerous studies have recently probed the relationships between m6A methylation and HCC pathogenesis. Wang et al. demonstrated that circ-KIAA1429 is expressed at a higher level in HCC cells than in normal cells, and the patients generally have shorter survival times (Wang M. et al., 2020). In addition, upregulated circ-KIAA1429 can be found in node metastasis status. These results indicate the fact is that KIAA1429 serves as an oncogene to further HCC invasion and migration by altering the methylation of m6A in *ID2* and *GATA3* mRNA (Lan T. et al., 2019; Cheng et al., 2019). Previous evidence revealed that Zeb1 was considered to be the downstream target of KIAA1429. Meanwhile, YTHDF3 is able to increase the stability of *Zeb1* mRNA, which participates in HCC tumorigenesis. The lifetime of Zeb1 gain improved via the effect of m6A modification (Wang M. et al., 2020). It was reported that circ-KIAA1429 contributed to the growing of invasion and metastasis process in HCC together with the mechanism of m6A-YTHDF3-Zeb1. Chen et al. demonstrated that elevated expression of ALKBH5 can be seen as a critical suppressor to impede proliferation and invasion of HCC by regulating the downstream target LYPD1. In HCC, LYPD1 is considered the oncogene that triggers the physiological process. Silencing of LYPD1 impairs growth and invasion of HCC. ALKBH5 is capable of modulating m6A modification and is involved in the IGF2BP1-associated pattern to regulate target LYPD1 (Chen et al., 2020).

**FIGURE 1:**
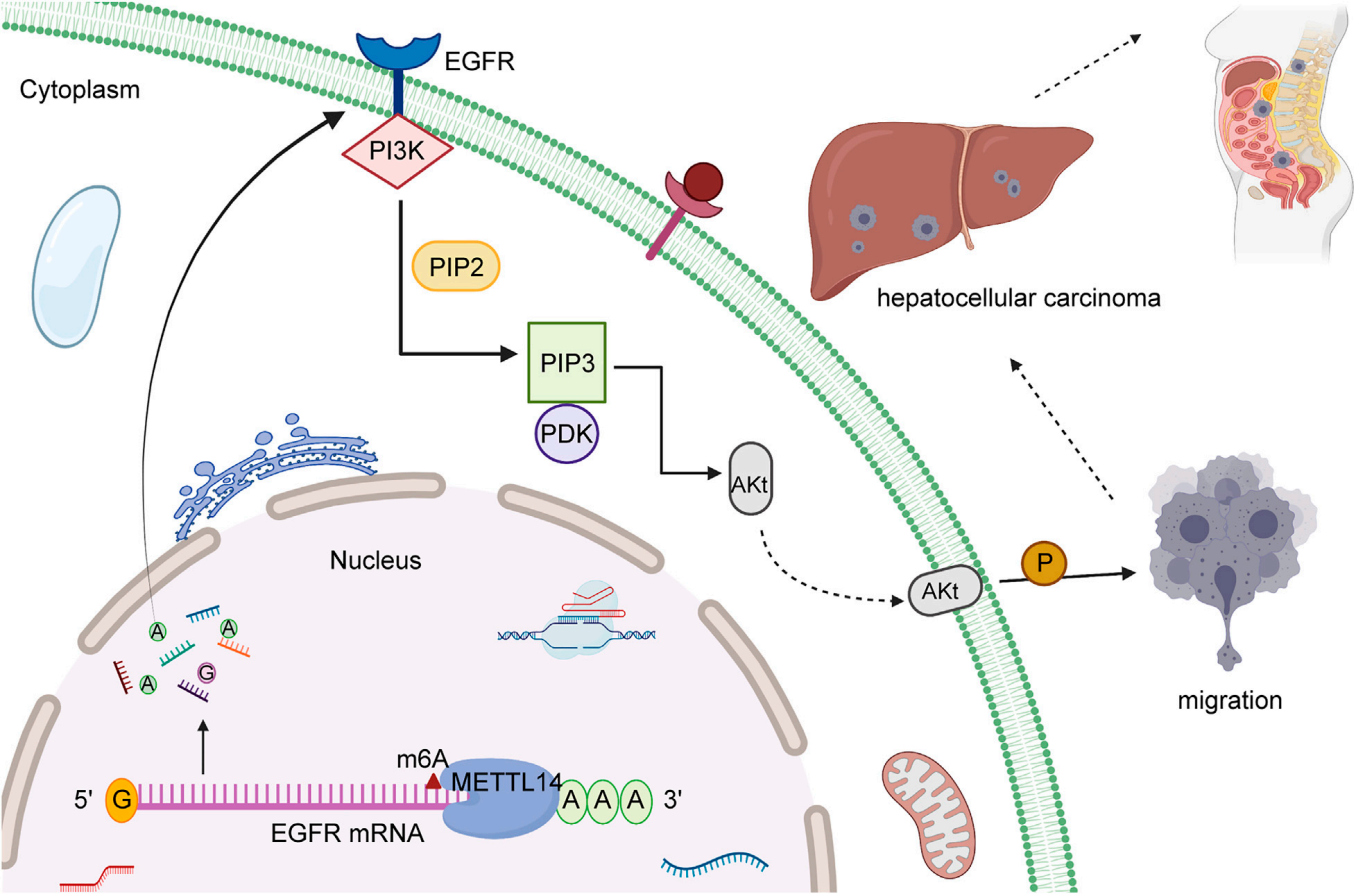
The molecular mechanism of METTL14 in HCC. METTL14, a “reader” of m6A methylation, can have a vital impact on inhibiting migration of HCC cells and is regarded as regulator to reduce EGFR mRNA stability through the effect of modification of m6A.

Previous survey unraveled HBXIP and METTL3 maintained high level in HCC patients. HBXIP could stimulate the occurrence of HCC cell malignant behaviors through the upregulation of METTL3 (Yang et al., 2021), catalyzing m6A methylation. METTL3 boosts HCC progression via post-transcriptional silencing of SOCS2 (Chen et al., 2018), whereas METTL3 knockdown reversed these effects by reducing m6A methylation. In contrast, METTL14 has been found to block the metastasis program of HCC, which decreases the stability of *EGFR* mRNA via posttranscriptional modification of m6A in Figure 1. EGFR was reported to play a critical role in the pathogenesis of various malignant tumors, such as in breast, pancreatic, prostate, colorectal, and liver cancer. In HCC, EGFR has been confirmed to stimulate the PI3K-AKT signaling pathway and foster the invasive and metastatic capacity of cells. These evidences suggested that EGFR might have the potential to become the promising target for treatment of HCC. Consequently, upregulated METTL14 effectively prevents migration of HCC cells, and is associated with positive prognostic outcome in a majority of patients. The suppressive property of METTL14 was revealed in a number of experiments (Shi Y. et al., 2020). Li et al. displayed that YTHDF1, a “reader” of m6A methylation, is upregulated in patients related to HCC and ordinarily is associated with dismal prognosis. HIF-1α interacts with YTHDF1 promoters, and upregulation of YTHDF1 was observed in a HIF-1α dependent manner. HIF-1α has been widely identified to trigger the transcriptional target gene to respond to hypoxic stress. HIF-1α avoids enzymatic degradation during hypoxic stress (Li Q. et al., 2021). YTHDF1 expression, mediated by HIF-1α, supports that hypoxic stress might lead to the alteration of cancer epigenetics, such as the translation of m6A-modified oncogenic mRNAs, to facilitate HCC malignancy. A recent study corroborated that AMD1 expression is the independent factor for overall survival (OS) and disease-free survival (DFS) in HCC (Bian et al., 2021). Several investigations found high expression of AMD1 in HCC tissue and showed that AMD1 regulates the expression of NANOG, SOX2, and KLF4, which are involved in HCC initiation, metastasis, and chemoresistance. Nevertheless, knockdown of AMD1 might increase the sensitivity of HCC cells to sorafenib. Previous report illustrated that FTO can promote the transcription of gene through the effect of removing numerous m6A modifications in the positions of 5′-UTR and CDs. Upregulated FTO could restrain the effect of down-expressed AMD1. While FTO presents the condition of silencing, the effect of AMD1 overexpression will be reversed. As a result, FTO severe as the downstream target of AMD1, and avails the therapeutic advancement for HCC.

### m5C Related to HCC

**FIGURE 2:**
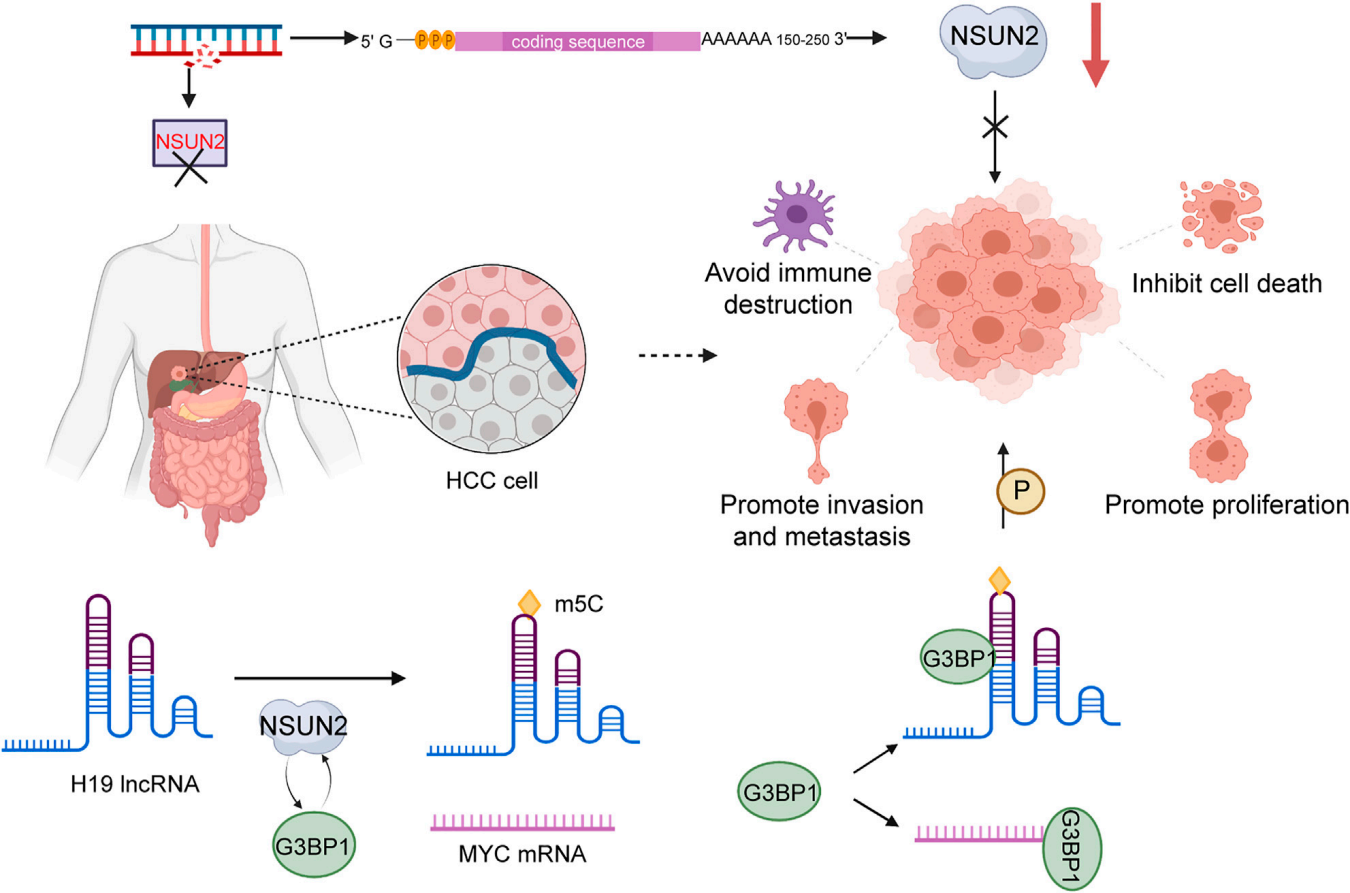
The role of NSUN2 in HCC. NSUN2 catalyzes m5C methylation in H19 lncRNA and enhances the malignant potential of HCC by promoting the binding of G3BP1 and H19.

A recent survey demonstrated that m5C modification has effects on distribution in HCC tissues and normal tissues. Compared with adjacent non-tumor tissue, high expression of m5C was shown in HCC tissue, which indicated that m5C methylation is closely associated with HCC pathogenesis (He et al., 2020a). NSUN2, a methyltransferase mediating the modification m5C, was confirmed to be upregulated in a variety of tumors in a previous study. Sun et al. showed that NSUN2 is clearly upregulated in HCC tissue have obvious upregulation of NSUN2 than normal tissues, and NSUN2 is capable of promoting the appearance of phenomenon about poor differentiation in HCC. Consistently, NSUN2 knockdown blocked the proliferation, invasion, and migration of HCC cells. Furthermore, NSUN2 have the property of stabilizing H19 by methylating H19 lncRNA. Overexpression of H19 is similarly found in HCC tissue with poor prognosis, and H19 is commonly seen as an important feature of poor differentiation in malignancy. Depletion of NSUN2 might give rise to cell inhibition in the G2 phase and prevent the increasing growth of HepG2 cells. Accumulating evidence demonstrates that the distribution of NSUN2 is variable during cell division; expression level is highest in S phase and lowest in G1. These results demonstrate that dynamic expression of NSUN2 has a profound impact on modulating cell division. NSUN2 catalyzed m5C methylation of H19 lncRNA to significantly affect malignant development of HCC. Consequently, H19 has the possibility of becoming a novel target of NSUN2. It was demonstrated that NSUN2 regulates m5C methylation of H19 lncRNA via interaction of Ras-GTPase–activating protein SH3 domain-binding protein 1 (G3BP1). G3BP1, a known oncoprotein that is generally expressed at a high level in multiple cancers that participate in diverse carcinogenesis-associated pathways containing Ras/MAPK (Liu S.-Y. et al., 2016), Wnt/β-catenin, PI3K/AKT (Zhang et al., 2019), and NF-κB/Her2 signaling pathways. These pathways could be regulated by NSUN2 through involvement in G3BP1 binding to H19 lncRNA (Sun et al., 2020), playing an essential role in malignant progression of HCC. In addition, G3BP1 also binds *MYC* mRNA to advance the effect of degradation (Tourrière et al., 2001). H19 lncRNA promotes tumor proliferation by binding G3BP1 and competing with *MYC* mRNA. When H19 lncRNA is poorly methylated, binding to G3BP1 will be further attenuated. Interestingly, MYC was found to accelerate H19 lncRNA transcription (Barsyte-Lovejoy et al., 2006). Therefore, the MYC-NSUN2-H19-G3BP1 axis was revealed to be associated with malignant behaviors of HCC (Figure 2). Moreover, the methylation modification could bring the decline number of circRNA, resulting in lack of suppression from crucial proteins and inducing the initiation of tumors (He et al., 2020b).

### m1A Associated With HCC

As a burgeoning discussion hotspot, research on m1A modification links to multiple cancers is also gradually becoming the basis of extensive concern. The understanding of m1A function related to HCC still requires further exploration. Shi et al. illustrated that TP53 mutations were primarily correlated with regulators mediating m1A methylation (Shi Q. et al., 2020). TP53 is a suppressor of various malignancies. However, the occurrence of TP53 mutations rapidly promotes tumorigenesis; for example, TP53 mutations serve as prognostic indicators of short survival time in HCC. Additionally, m1A-associated regulators expression actively has the impact on promoting progression of high TNM stage, including expression of RMT6, TRMT61A, TRMT10C, and TRMT6. It was reported YTHDF1 is valuable in predicting prognosis due to improving TRMT6 expression. Mounting survey unravel that m1A methylation might be regulated by the PI3K/Akt signaling pathway in HCC. The PI3K/Akt pathway plays a key role in proliferation and inhibition of apoptosis in HCC (Fu et al., 2019; Zheng et al., 2019). Nevertheless, how the PI3K/Akt pathway is involved in m1A and induces the development of HCC still needs further study. These findings suggest m1A has the potential to become a valuable biomarker in HCC.

## Conclusion

RNA methylation has emerged as the post-transcriptional modification to significantly affect a variety of genes expression processes, which not only has a broad influence on RNA metabolism but alters the function of various RNAs. Numerous proteins regulate methylation, demethylation, and specifically bind to diverse RNAs to promote or inhibit the biological functions, and are referred to, respectively, as “writers,” “erasers,” and “readers”. Prior research found aberrant expression of these regulators might lead to increasing disease. We summarize the distribution and functional consequences of m6A, m5C, and m1A modifications to further understand the role of RNA methylation and corresponding physiological mechanisms in HCC. For instance, overexpression of NSUN2 could promote malignant behaviors of HCC. METTL14, the “writer” of m6A, was proved to prevent metastasis of HCC. In this review, we found that RNA methylation may potentially serve as a novel marker and make valuable contributions to diagnosis and treatment in HCC, providing a promising future for a great many patients. Simultaneously, many studies are necessary to further explore and testify for clinical application.
